# Critical Roles of Reactive Oxygen Species in Age-Related Impairment in Ischemia-Induced Neovascularization by Regulating Stem and Progenitor Cell Function

**DOI:** 10.1155/2016/7095901

**Published:** 2015-11-30

**Authors:** Yuen Ting Lam

**Affiliations:** The Heart Research Institute, 7 Eliza Street, Newtown, Sydney, NSW 2042, Australia

## Abstract

Reactive oxygen species (ROS) regulate bone marrow microenvironment for stem and progenitor cells functions including self-renewal, differentiation, and cell senescence. In response to ischemia, ROS also play a critical role in mediating the mobilization of endothelial progenitor cells (EPCs) from the bone marrow to the sites of ischemic injury, which contributes to postnatal neovascularization. Aging is an unavoidable biological deteriorative process with a progressive decline in physiological functions. It is associated with increased oxidative stress and impaired ischemia-induced neovascularization. This review discusses the roles of ROS in regulating stem and progenitor cell function, highlighting the impact of unbalanced ROS levels on EPC dysfunction and the association with age-related impairment in ischemia-induced neovascularization. Furthermore, it discusses strategies that modulate the oxidative levels of stem and progenitor cells to enhance the therapeutic potential for elderly patients with cardiovascular disease.

## 1. Introduction

Reactive oxygen species (ROS), such as superoxide anions (O_2_^∙−^) and hydrogen peroxide (H_2_O_2_), are generated as electrons “leak” and react with oxygen molecule (O_2_) during mitochondrial oxidative phosphorylation. Alternatively, the formation of intracellular ROS can be catalyzed by an enzymatic reaction, where NADPH oxidase (Nox) transfers an electron to O_2_ and generates O_2_^∙−^. Aging is associated with increased oxidative stress that is characterized by an unbalanced redox homeostasis when the rate of ROS formation exceeds the capacity of endogenous antioxidative system to remove ROS. “Free Radical Theory of Aging” proposes that the production of ROS causes an accumulation of cellular damage, including DNA, proteins, and lipids, leading to a decline in mitochondrial integrity. This, in turn, drives a vicious cycle of ROS formation and exacerbates cellular damage, contributing to cellular senescence and premature aging [1]. This theory is supported by numerous studies using a wide range of model organisms, such as* Saccharomyces cerevisiae, Drosophila melanogaster, Caenorhabditis elegans, *and rodents, demonstrating a strong correlation between increased levels of ROS and oxidatively damaged molecules as cells aged [2–6]. An abnormal elevation of intracellular ROS also has an implication in pathogenesis of various diseases, such as ataxia telangiectasia and Fanconi anemia [7]. Nevertheless, ROS are important for cell signaling and homeostasis. “Redox window” hypothesizes that while excessive ROS contribute to the pathological conditions, appropriate ROS production from mitochondrial oxidative phosphorylation and NADPH oxidase is required for normal physiological responses [8].

**Figure 1:**
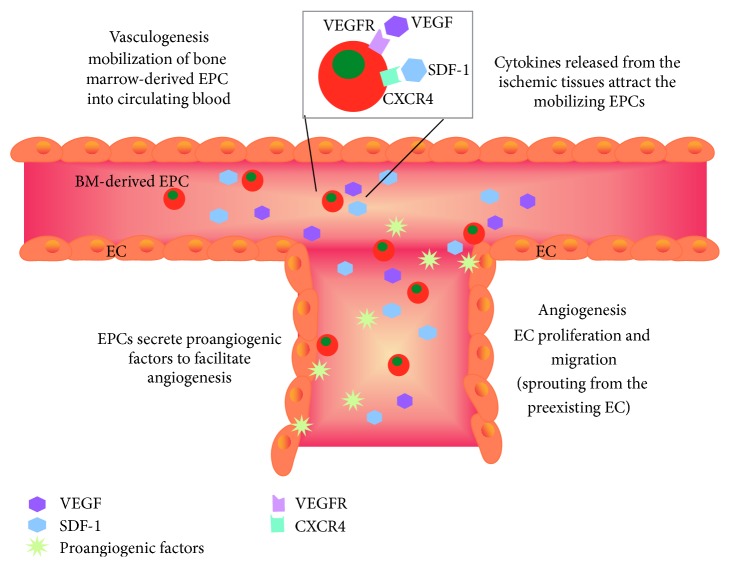
Schematic diagram of mechanisms involved in ischemia-induced neovascularization. Ischemia induces angiogenesis, the sprouting of new blood vessels from the preexisting ones. It involves the proliferation and migration of endothelial cells (ECs) at the local ischemic tissues. Cytokines, such as vascular endothelial growth factor (VEGF) and stromal cell-derived factor-1 (SDF-1), are released from the ischemic tissues to facilitate the recruitment of mobilizing endothelial progenitor cells (EPCs) through the binding of receptors. Vasculogenesis involves the proliferation and mobilization of EPCs from bone marrow to the circulating blood. EPCs express various surface receptors, such as VEGF receptor (VEGFR) and C-X-C motif receptor 4 (CXCR4). Once home to the ischemic sites, EPCs are capable of integrating with ECs and promote angiogenesis by secreting proangiogenic factors.

Cardiovascular disease is a major cause of world-wide mortality. Aging alone, without any other clinical manifest conditions, is a risk factor for coronary and peripheral artery diseases [9]. The majority of cardiovascular disease-related deaths are elderly individuals aged 75 and older. Following ischemia, vascular system is capable of repair and regeneration. The formation of new blood vessels (postnatal neovascularization) relies on two processes: (i) angiogenesis, the sprouting of mature endothelial cells from the preexisting vessels, and (ii) vasculogenesis, the mobilization of bone marrow-derived endothelial progenitor cells (EPCs) to the circulation (Figure 1). Aging is associated with impaired ischemia-induced angiogenesis and vasculogenesis* in vivo* [10–12]. EPCs are a subpopulation of progenitor cells originating from stem cells that differentiate into various lineage-committed cells. Although studies have used different markers to identify EPCs or referred to different nomenclatures, such as bone marrow-derived angiogenic cells, circulating progenitor cells, or proangiogenic myeloid cells [13–15], it is acknowledged that there is an age-dependent exhaustion of EPC numbers and/or impairment in EPC functions (Table 1).

This review summarizes current understanding of the involvement of (i) redox regulation in self-renewal, differentiation, and senescence of stem and progenitor cells; (ii) ROS as signaling molecules to mobilize progenitor cells from bone marrow to the circulation in response to ischemia; and (iii) how oxidative stress plays a role in age-dependent impairment in ischemia-induced neovascularization. With an increase in global aging population, a major concern is to understand the mechanistic role of age-related impairment in neovascularization in an attempt to develop better cell-based therapeutic strategies for elderly patients with vascular diseases.

## 2. The Role of ROS in Maintaining Stem Cell in Bone Marrow Microenvironment

Stem cells reside in a specialized bone marrow microenvironment (niche) [54]. Hematopoietic stem cells (HSCs) are one of the most characterized adult stem cells, which differentiate into all types of immune cells and maintain blood production. HSCs are predominantly located in hypoxic endosteal niche of the bone marrow with low-oxygen tension where a protection from ROS-related oxidative stress is provided [55, 56]. Jang and Sharkis 2007 have demonstrated that lineage depleted, CD45+ viable cell population (Lin−/CD45+/AnV−) could be separated into two fractions based on intracellular ROS levels, indicated by a fluorescence probe 2′-7′ dichlorofluorescein diacetate (DCF-DA). The levels of intracellular ROS correlate with stem cell capacities in self-renewal and differentiation. The isolated ROS^low^ population displays self-renewal ability by expressing higher levels of telomerase compared to ROS^high^ population [57]. Telomerase activity has been reported to be associated with the self-renewal potential of HSCs in mice [58]. On the other hand, the expression of a cyclin-dependent kinase inhibitor, p16InK4a, is upregulated in ROS^high^ population. As a biomarker of aging, p16Ink4a expression is found increased in most of rodent tissues with advancing age [59]. The accumulation of p16Ink4a levels is also associated with decreased repopulating activity and self-renewal abilities of HSCs in the older mice [60]. Furthermore, ROS^high^ population exhibits an increase in p38/mitogen-activated protein kinase (MAPK) activation. Elevation of ROS induces phosphorylation of p38/MAPK, which has been reported to limit self-renewal function in HSCs [61]. The reduction of self-renewal ability in ROS^high^ population can be restored by suppressing ROS production or ROS-induced p38/MAPK activation with antioxidant N-acetyl-L-cysteine (NAC) or p38 specific inhibitor [57].

**Figure 2:**
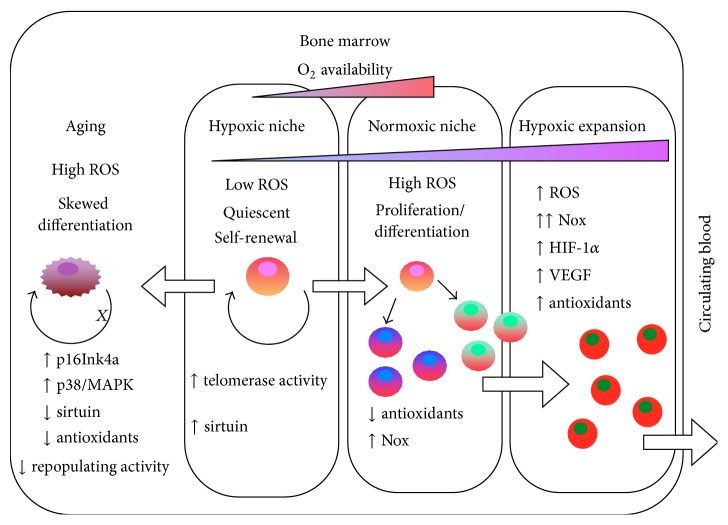
Schematic diagram of bone marrow microenvironment (Niche). Stem cells with self-renewal capacity reside in the hypoxic niche where the levels of reactive oxygen species (ROS) are low. Proliferation and differentiation of stem cells occur at the oxygenic niche where higher levels of ROS promote cell differentiation. During ischemia, hypoxic expansion upregulates transcription factor and hypoxia inducible factor-1*α* (HIF-1*α*), increasing the levels of vascular endothelial growth factor (VEGF) expression in the bone marrow. Meanwhile, there is an increase in NADPH oxidase- (Nox-) mediated ROS production. With aging, stem cells loss their self-renewal ability and displayed a skewed differentiation pattern (for details please see text).

While low-oxygen niche that limits ROS production is required to maintain HSCs at quiescent state in the bone marrow, the more oxygenic vascular niche (due to the proximity to the blood circulation) is essential for the proliferation and differentiation of stem cells to become progenitor cells (Figure 2). Increased intracellular ROS levels are found during the early stages of embryonic stem cell differentiation. Low levels of H_2_O_2_ induce cardiomyogenesis of embryonic stem (ES) cell, stimulating the proliferation of ES cell-derived cardiomyocytes. Several antioxidative genes and stress resistance genes are downregulated during embryonic stem cell differentiation into embryoid bodies [62]. NADPH oxidase isoforms, Nox1, Nox2, and Nox4, are upregulated; as a result, there is a feed-forward regulation of ROS generation during ES differentiation. Inhibition of Nox-derived ROS abolishes ES cardiomyogenesis [63].

## 3. Ischemia-Induced ROS Mediate Stem/Progenitor Cell Proliferation and Mobilization

During early stages of hypoxia, there is a transient elevation of intracellular ROS formation, as detected in various isolated tissues, such as skeletal muscle [64], systemic vessels [65], and myocardium [66]. Hypoxia-induced ROS may be a part of normal physiological response to the imbalance in oxygen supply and demand. Demonstrated by* in vivo* injection of O_2_^∙−^ reactive dye, dihydroethidium (DHE), Urao et al. 2012 show that hindlimb ischemia induces ROS production in both the endosteal and central regions of the entire bone marrow* in situ*. In conjunction with the increase in ROS levels, hindlimb ischemia also induces hypoxic expansion in bone marrow microenvironment [67]. The spatial distribution of hypoxia in bone marrow is visualized by an* in vivo* injection of hypoxic bioprobe, pimonidazole, that detects area less than 1.3% O_2_ by cross-linking protein adducts at oxygen tension below 10 mmHg [68]. These changes in the bone marrow microenvironment lead to upregulation of hypoxia-inducible factor-1*α* (HIF-1*α*) and vascular endothelial growth factor (VEGF) throughout the bone marrow (Figure 2). The mechanisms of how a distal ischemia in the hindlimb is capable of inducing an increase in ROS and hypoxic expansion in the bone marrow are yet fully understood. Nevertheless, Nox2 deficiency abolishes ischemia-induced hypoxic expansion and HIF-1*α* expression in the bone marrow microenvironment. Moreover, the levels of circulating EPC-like c-kit+/Flk1+ cells or c-kit+/Lin− progenitor cells are decreased in Nox2−/− mice following hindlimb ischemia [67, 69]. Therefore, Nox2-derived ROS induces hypoxic expansion and HIF-1*α* expression in the bone marrow microenvironment, which plays a role in progenitor cell expansion and mobilization into the circulating blood following ischemia.

The components of NADPH oxidase are expressed in various stem and progenitor cells including human bone marrow-derived CD34+ cells [70, 71], mouse embryonic stem cells [63], skeletal muscle precursor cells [71], and rat mesenchymal stem cells [72]. The constitutively active NADPH oxidase generates low levels of H_2_O_2_ in HSCs, which in turn stabilizes HIF-1*α* expression by inhibiting prolyl hydroxylases- (PHD-) mediated degradation of HIF-1*α* under normoxic conditions. An increase in HIF-1*α* expression is found in granulocyte colony stimulating factor- (G-CSF-) mobilized CD133+ and CD34+ HSCs from the peripheral blood of healthy donors [71, 73]. ROS-mediated HIF-1*α* stabilization may offer an advantage of enhancing the proangiogenic and antioxidative potential of the mobilizing bone marrow HSCs prior to homing to the hypoxic tissues, thereby facilitating neovascularization and tissue repair.

Interestingly, populations of more committed progenitor cells are intrinsically less sensitive to the elevation of intracellular ROS levels compared to HSCs. Serial transplantation of human Lin−/CD34+/CD38− HSCs into immunodeficient mice triggers replicative stress-induced elevation of intracellular ROS and leads to HSC premature senescence due to persistent DNA damage. However, Lin−/CD34+/CD38+ progenitor cells are more resistance to oxidative DNA damage [74]. Human EPCs isolated from peripheral blood followed by a short term* ex vivo* culture (4 days) exhibit low intracellular levels of H_2_O_2_ and O_2_^∙−^. The expressions of antioxidative enzymes, such as manganese superoxide dismutase (MnSOD), catalase, and glutathione peroxidase, are higher in EPCs compared to mature endothelial cells (ECs). Furthermore, the intracellular levels of ROS remain stable when EPCs are exposed to a redox cycler, napthoquinolinedione, that generates H_2_O_2_ and O_2_^∙−^. EPCs are also less sensitive to ROS-induced apoptosis compared to mature endothelial cells [75]. In another study, He et al. 2004 reported that catalase and CuZnSOD enzymatic activities are similar between EPCs and mature ECs and that mitochondrial MnSOD is the most likely of the three antioxidants to be responsible for EPC resistance to oxidative stress [76]. The discrepancy in antioxidant upregulation between the two studies may be due to the methodology of EPC culture and the definition of EPCs. In Dernbach et al. study, EPCs were cultured for 4 days and are characterized by an uptake of acetylated low-density lipoprotein (Dil-Ac-LDL) and lectin [77]. On the other hand, EPCs were cultured for an extended period, 2-3 weeks, and displayed cobblestone phenotype in He et al. study. In fact, the term “EPCs” in He et al. study may be better described as late-outgrowth endothelial cells (OECs). Demonstrated by Sieveking et al. 2008, EPCs and OECs have distinctive differences in angiogenic properties [78]. Therefore, it is speculated that the levels of antioxidative defense may be fine-tuned depending on functions and status of progenitor cells. OECs may require a higher tolerance toward mitochondrial oxidative stress and maintain mitochondrial structure integrity, which lessens the need for cytosolic antioxidants compared to EPCs. Features of stem and progenitor cells are summarized in Table 2.

**Figure 3:**
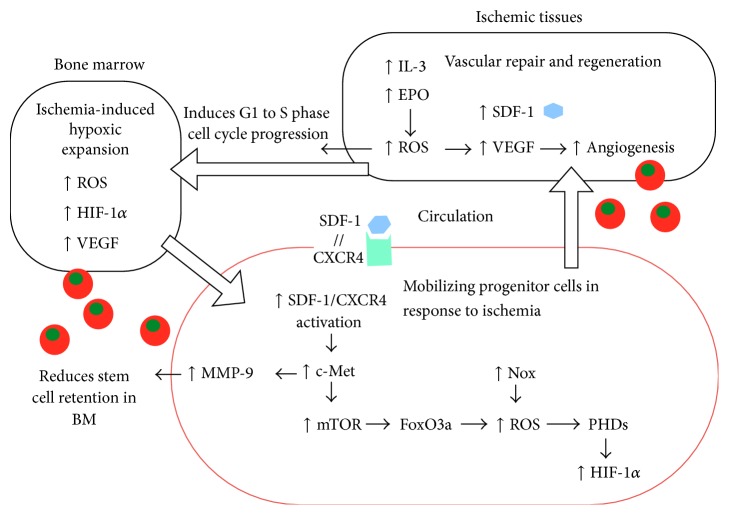
Schematic diagram of mechanisms involved in ischemia-induced progenitor cell mobilization. Under ischemic conditions, stromal cell-derived factor-1 (SDF-1) and hematopoietic cytokines, such as interleukin-3 (IL-3) and erythropoietin (EPO), are released from the tissues. Hematopoietic cytokines increase ROS and induce G1 to S phase cell cycle progression. Meanwhile, ischemia induces hypoxic expansion in the bone marrow to promote cell proliferation and differentiation (see Figure 2). In the circulation, SDF-1 binds to progenitor cells (outlined as red) expressing its receptor, CXCR4. SDF-1/CXCR4 activation induces c-Met and mTOR leads to downregulation of FoxO3a and increase in ROS production. Activation of c-Met upregulates the expression of matrix metalloproteinase-9 (MMP-9), which inhibits the adhesive interaction of progenitor cells to bone marrow (BM). In addition, NADPH oxidase (Nox) promotes ROS formation, which in turn stabilizes the levels of hypoxia inducible factor-1*α* (HIF-1*α*) by inhibiting prolyl hydroxylases (PHD). The mobilized progenitor cells facilitate the vascular repair and regeneration at the ischemic tissues.

ROS generation from the ischemic tissues also plays a role in promoting stem and progenitor cell mobilization. For example, ischemic skeletal muscles increase the production of hematopoietic cytokines, such as interleukin-3 and erythropoietin, which induce a rapid and transient ROS production. Subsequently, these cytokines promote bone marrow progenitor cells exiting quiescence through G1 to S cell cycle progression [79]. Stromal cell-derived factor-1 (SDF-1) released in ischemic tissue promotes stem and progenitor cell mobilization into the circulation by binding to C-X-C motif receptor 4 (CXCR4) [80]. SDF-1-induced chemotaxis is regulated by c-Met activation [81], which is known to control complex biological program of “invasive growth” and tumor spreading [82]. Activation of c-Met induces mTOR signaling and downregulates the levels of FoxO3a, which belong to the Forkhead Box, class O (FoxO) family of transcription factors that regulates oxidative stress. As a result, c-Met activation promotes the G-CSF-induced mobilization of bone marrow primitive progenitor cells (Sca1+/c-Kit+/Lin−) via increasing levels of ROS and upregulates the expression of proteolytic enzyme matrix metalloproteinase-9 (MMP-9) [81]. MMP-9 enhances progenitor cell mobilization into the circulation by inhibiting adhesive interaction necessary to retain stem and progenitor cells within the bone marrow [83] (Figure 3). Although it is beyond the scope of this review, low levels of ROS generated in the tissues during ischemic injury also promote local angiogenesis. The expressions of many angiogenic genes, such as VEGF, fibroblast growth factor, platelet-derived growth factor, and receptors are likely to be regulated by redox signaling [84]. Nox2-derived ROS is involved in VEGF signaling, which plays an important role in EC migration and proliferation, as well as reparative angiogenesis in response to hindlimb ischemia* in vivo* [85, 86].

## 4. Stem and Progenitor Cell Aging

With aging, stem cells lose self-renewal activity and terminally differentiated, thereby exiting the stem cell pool. On the other hand, they may undergo apoptosis or senescence induced by higher levels of ROS (Figure 2). Age-related depletion of stem cell pool may be driven by an imbalance of intracellular ROS that regulates stem cell quiescence and proliferation. Aged mice have a decrease in ROS^low^ population of Lin−/CD34+/AnV− cells, indicating a reduction of HSC populations that are capable of more durable long-term self-renewal [57]. HSCs from elderly individuals also exhibit higher levels of ROS and have reduced ability to reconstitute hematopoiesis of murine host compared with HSCs from middle-aged individuals [74]. Aged HSCs display a skewed differentiation potential, in which these cells overproduce myeloid lineage cells rather than a multilineage population, consisting of both myeloid and lymphoid lineage cells [57, 60, 87].

The sirtuin family of NAD-dependent deacetylases, a key regulator of organismal longevity, has been shown to modulate stem cell aging. Deletion of SIRT1 in young HSCs displays an aging phenotype with a skewed differentiation toward myeloid lineage that is associated with a decline in lymphoid compartment [88]. Lentiviral shRNA knockdown of SIRT1 in human bone marrow-derived mesenchymal stem cell accelerates cellular senescence [89]. Recently, a link between oxidative metabolism and sirtuin in modulating stem cell homeostasis has been reported. Brown et al. 2013 show that SIRT3 is downregulated with age and is accompanied with a reduction of mitochondrial MnSOD activity which in turn contributes to increased ROS levels in aged HSCs. Demonstrated by an* in vivo* competitive transplantation assay, it is showed that SIRT3 is required to maintain HSC pool size and regenerative capacity under oxidative stress conditions, such as aging and serial transplantation [90]. SIRT3 preserves HSC functions by enhancing mitochondrial MnSOD antioxidative activity via posttranslational deacetylation of critical lysine residues [90, 91]. Furthermore, SIRT3 has a critical role in bone marrow cell-mediated cardiac repair. It is shown that intramyocardial injection of bone marrow cells from SIRT3 knockout mice results in reduced numbers of Sca1+/c-kit+ progenitor cells mobilized to the ischemic area following myocardial infarction. The loss of SIRT3 increases ROS formation and cellular apoptosis, reducing the proangiogenic capability in EPCs* in vitro* [92].

Bioactive peptides in the vascular system also play a role in progenitor cell aging. Angiotensin II (Ang II) is a key effector of the renin-angiotensin system. Ang II not only regulates blood pressure as a potent vasoconstrictor, but also promotes inflammation, hypertrophy, and fibrosis. Ang II plays a role in vascular damage and remodeling in cardiovascular diseases [93–95]. It has been demonstrated that inhibition of angiotensin-converting enzyme by Ramipril augments circulating EPCs with enhanced functional activity in patients with stable coronary artery disease [96]. In human EPCs, Ang II increases the expression of Nox2 component, gp91phox, and accelerates the onset EPC senescence through an induction of oxidative stress, as evidenced by peroxynitrite formation* in vitro*. Pretreatment of EPC with SOD prevents Ang II-induced telomerase inactivation [97]. Hepatocyte growth factor (HGF) also attenuates Ang II-induced EPC senescence by reducing gp91phox expression and limiting the production of O_2_^∙−^ in EPCs [98].

Excessive production of ROS in pathological conditions has been associated with HSC exhaustion. “Ataxia telangiectasia mutated” (Atm) gene is responsible for genomic stability in response to DNA damage and oxidative stress. Mice with Atm deficiency (Atm−/−) exhibit progressive bone marrow failure in association with increased ROS levels [99]. The elevation of ROS induces p38/MAPK phosphorylation in HSCs and is accompanied by a defect in maintaining HSC quiescence. Treatment of Atm−/− mice with antioxidant NAC restores the HSC reconstitutive capacity and prevents bone marrow failure [61]. Loss of FoxO3a in HSCs also results in elevated oxidative stress, increased p38/MAPK phosphorylation, and defective maintenance of quiescence. The ability of HSCs to support long-term reconstitution of HSC pool in a competitive transplantation assay is impaired in FoxO3a−/− mice [100]. Conditional deletion of FoxO1, FoxO3a, and FoxO4 in mouse hematopoietic system leads to increased ROS levels, myeloid lineage expansion, and a reduction in Lin−/Sca1+/c-kit+ HSC population [101]. Indisputably, excessive ROS production is associated with the disruption of HSC quiescence and impairment in hematopoietic repopulating ability of HSCs in the bone marrow.

## 5. Unbalanced ROS Levels and Age-Related Impairment in Ischemia-Induced Neovascularization

The importance of balanced ROS levels in mediating ischemia response and neovascularization has been demonstrated in animal models manipulated to have impaired cellular antioxidant mechanisms. Heterozygous knockout of mitochondrial MnSOD results in an increased lipid peroxidation and a defect in myocardial contractile function followed by ischemia-reperfusion injury in isolated heart, while deletion of a single copy of cytosolic CuZnSOD does not show any significant impairment [102]. This suggests that there is a tissue-specific requirement of antioxidative enzyme in the tolerance to oxidative stress that cannot be compensated by the other SOD isoforms. Studies have also demonstrated a differential effect of aging on ROS-mediated ischemic response. Carotid arteries from young heterozygote CuZnSOD (+/−) knockout mice show no significant alternation in endothelial relaxation in response to acetylcholine. However, aged CuZnSOD (+/−) mice are impaired in endothelial-dependent vasodilation in response to acetylcholine, which can be restored by the presence of superoxide scavenger, tempol [103]. The effect of a single deletion of CuZnSOD is minimal in young age. ROS seem to be well tolerated in young cells with highly proficient antioxidative defense, which may be compensated by the remaining copy of CuZnSOD and other antioxidative enzymes. With aging, cells with less efficient antioxidative defense become sensitive to oxidative stress, which aggravates ROS formation. Eventually, aged cells are inadequate to maintain proper vascular functions. On the other hand, young homozygous CuZnSOD (−/−) knockout mice exhibit an accelerated vascular aging and impaired ischemia-induced neovascularization. Young CuZnSOD−/− mice have similar oxidative stress levels in the ischemic tissues as those observed in older wild type littermate, examined by immunofluorescence staining of DHE and nitrotyrosine (an indicator of protein nitration by ROS). Aged CuZnSOD−/− mice have the highest level of oxidative stress and display severe necrosis and autoamputation in the second weeks after surgically induced hindlimb ischemia [104]. CuZnSOD deficiency leads to increased ROS levels and is associated with reduced proangiogenic functions of EPCs, such as migratory ability and integration into endothelial cell tubules* in vitro* [105]. The peripheral EPC levels in the spleen are lower in both young and aged CuZnDOS−/− mice [104]. Authors suggest that CuZnSOD deficiency may cause a depletion of EPC reserve in the bone marrow and result in the impaired EPC mobilization observed in the spleen of CuZnSOD−/− mice. However, EPC levels in the bone marrow of CuZnSOD−/− mice and whether CuZnSOD deficiency attenuates EPC mobilization from the bone marrow to the circulating blood remained to be determined. Nevertheless, aging exacerbates oxidative stress-associated EPC dysfunction in the absence of CuZnSOD. CuZnSOD has a critical role in limiting excessive ROS accumulation and preserves EPC angiogenic activities with aging. The role of CuZnSOD in modulating EPC numbers and functional activities may also have important clinical implications. Patients with chronic heart failure and coronary artery disease have lower levels of antioxidant enzymes, including CuZnSOD [106, 107], which may explain reduced EPC numbers and functions in patients [23, 108]. Alternatively, protection against age-dependent impairment in ischemia-induced neovascularization in association with excessive ROS formation has been demonstrated in Nox2 deficient mice. Nox2 deficiency ameliorates age-related increase in ROS levels and enhances bone marrow-derived EPC proangiogenic functions* in vitro.* As a result, aged Nox2−/− mice exhibit enhanced blood flow recovery following ischemia [109]. As the tolerance of oxidative stress decreases with age, it is essential to preserve stem and progenitor cell vasculogenic functions by maintaining ROS balance.

## 6. Therapeutic Potential of Cell-Based Therapy for Age-Related Impairment in Neovascularization by Modulating Redox Regulation

Implantation of autologous bone marrow-derived stem and progenitor cells is a potential treatment for ischemic diseases. Cell-based therapies have been safely conducted and demonstrated beneficial effects in augmenting neovascularization in preclinical animal studies (Table 3). Despite the promise of preclinical studies, human clinical trials involving an administration of autologous bone marrow cells or progenitor cells from bone marrow or peripheral blood have, to date, yielded neutral or underwhelming outcomes (Table 4). In experimental settings, stem and progenitor cells are often isolated from healthy young animals as donors and transplanted into young healthy recipients. The isolated stem cells are often highly regenerative and may contain less cumulative ROS-related damage. However, patients who would undergo cell-based therapy are elderly who have increased ROS levels and reduced numbers of stem and progenitor cells with impaired regenerative potential.

Studies have investigated different approaches to enhance the therapeutic potential of stem and progenitor cells by modulating their redox regulation. The first strategy involved suppressing excessive oxidative stress by promoting the antioxidative potential. For example, transgenic expression of MnSOD or administration of SOD mimic rescues impaired postischemia neovascularization and tissue survival in diabetic mice [110, 111]. Mesenchymal stem cell engraftment in the infarct heart is enhanced by coinjection of antioxidant NAC which mitigates ROS-induced inhibition of cell-matrix adhesion [112]. Intraperitoneal injection of SOD mimic reduces ROS formation and facilitates CD34+ progenitor cell recruitment to the infarct heart following coronary ligation in mice [113]. Preconditioning stem cells, with either a brief period of ischemia/anoxia or repeated cycles of intermittent hypoxia/reoxygenation, increase postengraftment cell survival or neovascular potential through oxidative stress resistance mechanism [114]. Electrical stimulation also provides preconditioning effect on the survival of cardiac stem cells and protects against oxidative stress-induced apoptosis via AKT activation by downregulating miR-378 [115]. Another approach involved stimulating stem cells with low-dose of prooxidants. Short-term treatment of mouse bone marrow cells with 5 *μ*M H_2_O_2_ for 30 min enhances their angiogenic potency by promoting VEGF production and endothelial differentiation [116].* In vitro* treatment of adipose-derived stroma cells (ADSCs) with pharmacological inhibitors to generate mitochondrial ROS increases the secretion of proangiogenic factors and protects ADSCs against ROS-induced apoptosis. Furthermore,* in vivo *injection of the treated ASCs promotes neovascularization in hindlimb ischemia [117]. Although most of these enhancements are demonstrated in cells from young donors and recipients, some have been shown effective in aged cells and old recipients. For example, preconditioning of bone marrow cells from aged mice (20–22 months old), by culturing cells at 2% O_2_ for 24 hours, shows enhanced adhesion, survival, and proangiogenic potential* in vitro. *The preconditioned cells augment ischemia-induced neovascularization in aged mice following intramuscular injection [118]. Hypoxic preconditioning of human ADSCs from donor over 50 years old at 0.5% O_2_ for 24 hours increases redox metabolism and promotes paracrine secretion [119]. Treatment of bone marrow-derived angiogenic cells from aged mice (17 months old) with dimethyloxalylglycine (DMOG), an *α*-ketoglutarate antagonist, induces HIF-1*α* that leads to metabolic reprogramming and decreases ROS formation in these aged cells. In combination with HIF-1*α* gene therapy in the ischemic muscle tissues, intravenous injection of DMOG-treated cells prevents limb necrosis and autoamputation in old recipients mice following ischemia [120]. While there are many strategies that modulate the redox regulation of stem and progenitor cells, it is essential for* in vitro* and preclinical studies to consider the clinical scenario, where elderly patients often suffer from comorbidities that affect neovascularization, such as diabetes, hypercholesterolemia, and advanced atherosclerosis. Therefore, it may be beneficial to develop cell-based therapies targeting combined pathophysiological conditions such as aging, metabolic disorders, and inflammatory diseases.

## 7. Conclusion

ROS production and aging are intertwined biological events that play a critical role in vascular repair and regeneration. ROS are intrinsic regulators that are involved in maintaining the abilities of self-renewal of stem cells and their differentiation into “lineage-committed” progenitor cells. The levels of ROS are attuned by the balance between ROS generation and antioxidative defense systems, depending on the cellular functions at different stages of stem and progenitor cells. On the other hand, ROS are extrinsic mediators that modulate bone marrow microenvironment in low-oxygen tension and induce hypoxic expansion in response to ischemic injury. Aging is associated with an increase in oxidative stress, in which the unbalanced ROS levels further contribute to cell aging. The age-dependent impairment in ischemia-induced neovascularization is, partly, due to oxidative stress-related dysfunction of stem and progenitor cells. Understanding the molecular targets of ROS and distinct redox signaling pathways in stem and progenitor cell function as well as how aging alters the redox balance will enable us to improve the efficacy of cell-based therapies and to better accommodate cardiovascular disease in aging populations.

## Figures and Tables

**Table 1:** Examples of age-dependent exhaustion of EPC numbers and reduction of EPC functions.

Study	Subjects	Source of cells	EPC markers	Effect of aging	Reference
Rauscher et al. 2003	6-month-old *versus* 1-month-old ApoE−/− mice	BM	CD31+/CD45−	Reduced EPC numbers; progressive development of atherosclerosis	[16]
Zhang et al. 2006	12-month-old *versus* 3-month-old BALB/C mice	BM	CD117+/CD34+/Flk1+	Decrease in numbers; reduced EPC proliferation, migration, and phagocytic functions	[17]
Sugihara et al. 2007	18-month-old *versus* 2-month-old C57Bl/6J mice	BM	AC133+/CD34+ and CD34+/VEGFR2+	No difference in numbers of EPCs; impaired VEGF production and EPC migration	[18]
Shimada et al. 2004	*Klotho *mutant^*∗*^ * versus* wild type mice	BM and peripheral blood	c-kit+/CD31+ and CD34+/CD31+	Decrease in EPC numbers postischemia	[19]
Chang et al. 2007	18–24-month-old *versus* 4–6-month-old C57Bl/6J	Peripheral blood and BM	CD11b−/Flk1+ and Sca1+/c-kit+/Lin−	Decrease in CD11b−/Flk1+ numbers in blood, but not Sca1+/c-kit+/Lin− cells in BM postischemia	[11]
	68–95-year-old *versus* 18–35-year-old human	Peripheral blood	AC133+	No difference in EPC numbers at baseline	
Zhuo et al. 2010	15-16-month-old *versus* 2-month-old rat	Peripheral blood and spleen	CD34+/KDR+	Decreased numbers in response to ischemia, but not at baseline (prior to ischemia)	[20]
Shao et al. 2011	24–26-month-old *versus* 2-month-old C57Bl/6J mice	BM	Lin−/Sca1+ and Lin−/Sca1+/CXCR4+	Decrease in Lin−/Sca1+/CXCR4+, but not Lin−/Sca1+ subpopulation	[21]
Boon et al. 2011	16–18-month-old *versus* 1-month-old C57Bl/6J mice	Peripheral blood	Lin−/Sca1+/c-kit+, Sca1+/c-kit, and Sca1+/Flk1+	Decrease in all 3 populations	[22]
Scheubel et al. 2003	Patients with CAD; 69 years old *versus* younger patients	Peripheral blood	AC133+/CD34+	Reduced basal circulating EPC levels	[23]
Heiss et al. 2005	Healthy elderly (average 61 years old) *versus* healthy young subjects (average 25 years old)	Peripheral blood	CD133+/KDR+ and CD34+/KDR+	Comparable levels of EPCs	[24]

BM, bone marrow.

CAD, coronary artery disease.

^*∗*^ *Klotho* mutant mice, an animal model of typical aging, display accelerated arteriosclerosis.

**Table 2:** Summary of distinctive features of stem and progenitor cells.

Cell type	Features and functions
Hematopoietic stem cells (HSCs)	
Lin−/CD34+/AnV−	Lineage depleted, viable (annexin negative), undifferentiated, and primitive multipotential hematopoietic stem cells
	ROS^low^: self-renewal, ↑telomerase
	ROS^high^: limited self-renewal, ↑p16Ink4a, ↑p38/MAPK
	
Lin−/CD34+/CD38−	Lineage depleted, undifferentiated, and primitive multipotential hematopoietic stem cells
	ROS sensitive Serial transplantation of Lin−/CD34+/CD38− leads to DNA damage and premature senescence
	
Lin−/CD34+/CD38+	Lineage depleted, primitive hematopoietic/lymphoid stem cells Less sensitive to ROS elevation and resistant to oxidative DNA damage
	

Scal+/c-kit+/Lin−	Primitive stem cells Mobilized into circulation via increasing ROS in response to ischemia

Embryonic stem cells (ES)	
	Active in proliferation and differentiation
	Require low levels of H_2_O_2_ to trigger cardiomyogenesis
	↑Nox1, ↑Nox2, and ↑Nox4
	↓Antioxidative and stress resistance genes

Progenitor cells	
Endothelial progenitor cells (EPCs)	*Ex vivo* cultured for 4 days ↑MnSOD, ↑catalase, and ↑glutathione peroxides Stable intracellular ROS levels Resistance to ROS-induced apoptosis
	
Late-outgrowth endothelial cells (OECs)	*Ex vivo* cultured for 2-3 weeks ↑MnSOD Similar levels of CuZnSOD and catalase compared to mature endothelial cells

**Table 3:** Selected cell-based preclinical studies.

Cell treatment	Ischemic model	Outcomes	References
*Ex vivo* culture expanded human EPCs from healthy young individuals	Myocardial ischemia in athymic nude mice	Increased neovascularization; increased capillary density; reduced infarct size; improved LV function after myocardial ischemia	[25]
Human peripheral blood MNC-derived CD14+ or CD14− EPCs	Hindlimb ischemia in athymic nude mice	Increased blood perfusion; increased capillary density	[26]
Human blood-derived CD34+ cells	Hindlimb ischemia in diabetic mice	Increased blood flow perfusion in diabetic mice, but not in nondiabetic mice	[27]
Human blood cord-derived CD34+ EPCs	Cerebral ischemia in mice	Accelerated neovascularization of infarct neuronal tissue; increased cortical expansion; increased neuronal regeneration; improved recovery of motor deficits	[28]
*Ex vivo *expanded human EPCs from peripheral blood followed by VEGF transduction	Hindlimb ischemia in athymic nude mice	Reduced limb loss; increased blood flow recovery after ischemia; increased EPC incorporation *in vivo*	[29]
Autologous EPCs from peripheral blood	Pulmonary hypertension in dogs	Improved pulmonary artery pressure, cardiac output, and pulmonary vascular resistance	[30]
Autologous EPCs from peripheral blood	Carotid denudation in rabbits	Accelerated reendothelialization; improved endothelial function	[31]
Autologous CD34+ EPCs from bone marrow	Acute myocardial infarction in macaques	Improved regional blood flow; increased capillary density in the peri-infarct region; improved cardiac function; increased VEGF and bFGF levels in peri-infarct region	[32]

MNC, mononuclear cell.

VEGF, vascular endothelial growth factor.

bFGF, basic fibroblast growth factor.

**Table 4:** Selected human cell-based clinical studies.

Conditions	Cell type	Therapy	Delivery methods	Outcome	Reference
Acute myocardial infarction	BMC	BOOST (randomized controlled)	Intracoronary injection	Improvement in LVEF at 6-month follow-up, but it failed to sustain the functional enhancement at 18-month and 5-year follow-ups	[33]
	BMC	REPAIR-AMI (randomized controlled)	Intracoronary infusion	Improved LVEF at 4-month follow-up; improvement of LV function sustained at 12-month follow-up and reduced major adverse CV events	[34–36]
	BMC	STEMI (randomized controlled)	Intracoronary infusion within 24 h administration	Reduced infarct size, but no significant improvement in LV function at 4-month follow-up	[37]
	BMC	ASTAMI (randomized controlled)	Intracoronary injection	No changes in LV end-diastolic volume or infarct size at 6-month follow-up	[38]
	BMC	BALANCE (controlled but nonrandomized)	Intracoronary infusion	Improved LV function, contractility, infarct size, haemodynamics, and exercise capacity at 12- and 60-month follow-up	[39]
	CD133+ progenitor cells	Small scale; nonrandomized	Intracoronary infusion	Improved LVEF at 4-month follow-up but increased incident of coronary events	[40, 41]
	CD133+ progenitor cells	Small scale; nonrandomized	Transplantation to peri-infract zone during CABG surgery	Improvements in myocardial viability and local perfusion; no adverse events at 6-month follow-up	[42]
	BMC and BM-derived CD34+/CXCR4+ progenitor cells	REGENT	Intracoronary infusion	Increased LVEF; no significant differences in absolute changes of LVEF between groups at 6-month follow-up	[43]
	BM-derived MSCs	Randomized controlled	Intravenous injection	Increased LVEF; improved global symptom at 6-month follow-up; MSCs traps in pulmonary passage in animal model	[44, 45]
	BMC and circulating blood-derived CD34+ progenitor cells	TOPCARE-AMI (randomized controlled)	Intracoronary infusion	Improvement in LVEF at 3-month follow-up; effect of BMC transplantation is greater than CPC; functional improvements sustained for 2 years	[46, 47]

Ischemic cardiomyopathy	Autologous skeletal myoblasts	MAGIC (randomized controlled)	Injection around the scar tissues	No significant improvement in global and regional LV function; an increase in arrhythmic events in treated patients	[48]

Chronic heart failure	Bone marrow cells	STAR	Intracoronary infusion	Improvements in LV function, exercise capacity, and oxygen uptake over a 5-year follow-up	[49]

Refractory myocardial ischemia	Bone marrow cells	(Randomized controlled)	Intramyocardial injection	Improvement of myocardial perfusion, angina severity, and quality of life at 3-month follow-up	[50]
	CD34+ progenitor cells	ACT34-CMI (randomized controlled)	Intramyocardial, transendocardial injection	Improvement in angina frequency and exercise tolerance	[51, 52]

Severe coronary artery diseases	Bone marrow cells	PROTECT-CAD (randomized controlled)	Endomyocardial injection	Improved LV function, exercise time, and NYHA functional class at 6-month follow-up	[53]

BMC, bone marrow cells.

LVEF, LV ejection fraction.

MI, myocardial infarction.

NYHA, New York Heart Association.

CABG, coronary artery bypass grafting.

MSC, mesenchymal stem cell.

## Abbreviations

Ang II: Angiotensin II
CuZnSOD: Copper-zinc superoxide dismutase
CXCR4: C-X-C motif receptor 4
DHE: Dihydroethidium
EPC: Endothelial progenitor cell
FoxO: Forkhead Box class O
G-CSF: Granulocyte colony stimulating factor
HIF-1*α*: Hypoxia-inducible factor-1*α*
HSC: Hematopoietic stem cell
MAPK: Mitogen-activated protein kinase
MnSOD: Manganese superoxide dismutase
NAC: N-Acetyl-L-cysteine
Nox: NADPH oxidase
SDF-1: Stromal cell-derived factor-1
SIRT: Sirtuin
VEGF: Vascular endothelial growth factor.
